# The Psychometric Properties of Translating Self-Efficacy Belief: Perspectives From Chinese Learners of Translation

**DOI:** 10.3389/fpsyg.2021.642566

**Published:** 2021-04-06

**Authors:** Yanxia Yang, Xinyu Cao, Xing Huo

**Affiliations:** ^1^School of Foreign Languages, Nanjing Agricultural University, Nanjing, China; ^2^School of Foreign Languages, Southeast University, Nanjing, China; ^3^School of Foreign Languages, Hunan University, Changsha, China

**Keywords:** translating self-efficacy, Chinese learners of translation, translation competence, scale development, translator education

## Abstract

Understanding learners’ translating self-efficacy belief helps predict their ability to cope and translation performance during their career. Despite this connection, the assessment of self-efficacy during learning has been largely overlooked in translation research. The purpose of this study was to develop and validate an instrument to examine belief in self-efficacy in a sample of Chinese translation learners. Scale items were collected and refined based on an expert-panel consensus opinion. Exploratory factor analysis and confirmatory factor analysis across two independent samples (Sample I = 193 and Sample II = 247) revealed and validated a three-dimensional structure: efficacy in internal competence, efficacy in psycho-physical competence, and efficacy in external competence. These findings provide supporting evidence for scale applications in educating translators.

## Introduction

The demand for quality translators has led to the rapid development of translator-training programs (Pym, 2011). For example, by the year 2018, China authorized 252 colleges and universities to offer a Master of Translation and Interpreting (MTI) program. A further 272 colleges and universities have also established Bachelor of Translation and Interpreting (BTI) programs (Tao, 2019). In addition to translator-training programs, translation courses are offered as part of language, culture, and applied linguistics programs in tertiary institutions (Venuti, 2017). This trend can be seen in Chinese universities, where translation courses are offered to language and non-language students who have a competent mastery of a language and who want to improve their translation competence. According to *Language Service Development in China: 2020* (Qu, 2002), language and non-language graduates are working as part of a translator community in the language service industry. Hence, the concept of translation learners in this study covers translation students, language students, and non-language students.

The psychology of translators is relevant to translation activities, where the underlying cognition, emotional, behavioral, and social factors are at play (Bolaños-Medina, 2016). One of the factors is self-efficacy (Bolaños-Medina, 2014). Self-efficacy is a cognitive factor that can influence performance behaviors and affective processes (Bandura, 1997). Prior language learning research suggests that self-efficacy can play an influential role in choice-making, strategies, and effort (Prat-Sala and Redford, 2012; Bruning et al., 2013; Carroll and Fox, 2017). Despite the stated benefits, self-efficacy has only recently been introduced into translator education research. It is closely related to self-confidence and is considered to be a sub-component of self-confidence (Haro-Soler, 2018). On this basis, Haro-Soler and Kiraly (2019) define translating self-efficacy as the confidence that translators have in their abilities to perform translation activities. The scattered documentation suggests that self-efficacy is associated with the motivations of translators, expertise, and performance (Atkinson, 2014; Bolaños-Medina, 2014; Araghian et al., 2018; Haro-Soler, 2019a). The performance-stimulating effect of self-efficacy sheds light on the importance of examining translating self-efficacy.

Although some self-efficacy scales are available and tested, for example, scales for language learners (Wang et al., 2014), interpreters (Lee, 2014), and translators (Bolaños-Medina and Núñez, 2018; Haro-Soler, 2018), there is no translating self-efficacy instrument for measuring student translators, language and non-language students in an English as a foreign language environment. Amendments and adaptations based on other pioneering work are required to best fit the population and environment.

The purpose of the present study is to develop and validate a translating self-efficacy instrument (TSE-C), taking Chinese student translators as the research sample. In the following sections, we first review the literature on the concept and measurement of self-efficacy, as well as the role of self-efficacy in translation for the sake of scale item generation. We then describe the research design, consisting of participant profiles, instrument development, and data collection procedures. The validating process is discussed to indicate the validity of the scale and consider the reliability of the instrument.

## Theoretical Perspectives

### Sources of Self-Efficacy

Self-efficacy is about an individual’s self-judgment of their capabilities to complete a specific task with the skills they possess (Bandura, 1997). It is a primary explanatory construct in social cognitive theory which holds that human behavior is strongly stimulated by self-influence. This construct can influence an individual’s motivation, decision-making, persistence in the face of difficulties, and performance (Bandura, 1995).

An investigation of the domain sources of self-efficacy provides a deeper understanding of self-efficacy. Self-efficacy is developed through four domain sources: *enactive mastery experience*, *vicarious experience*, *verbal persuasion*, and *physiological and emotional states* (Bandura, 1997). *Enactive mastery experience* is a students’ self-perceived ability to successfully perform a task based on previous attainments (Zhang and Ardasheva, 2019). Students interpret the attainments of their previous tasks and use the interpretations to develop beliefs of their capability to perform subsequent tasks (Dinther et al., 2011). The interpreting process has created a sense of self-efficacy. However, the sense of self-efficacy is often generated from experience in overcoming obstacles and difficulties through maintained effort and persistence (Bandura, 1997). Since the master experience can provide students with evidence that they can complete a task, it is often considered as a powerful way of creating a strong sense of self-efficacy. *Vicarious experience* is about observations of others’ successes and failures, which can be interpreted as models of one’s performance (Schunk and Hanson, 1985). By observing the success and failure of other people with similar ability levels, students can enhance their belief in their self-efficacy by confirming their ability to perform a task. *Verbal persuasion* relates to persuasive feedback and comments on performance. It is often used to examine the impact of self-efficacy on effort, affective states, and task choice (Bandura, 1997). Comments highlighting students’ abilities can enhance their self-efficacy belief, while comments focusing on students’ shortcomings may undermine their self-efficacy. *Physiological and emotional states* are related to a student’s ability to manage physical and emotional stress reactions during task performance. Stressful situations, complexities of activities, and bad mood states may impair self-efficacy belief. The four sources of self-efficacy demonstrate how a student’s belief in their self-efficacy can influence their performance.

### Dimensions of Self-Efficacy

According to Bandura (1997), self-efficacy belief consists of three dimensions: *magnitude*, *strength*, and *generality*. *Magnitude* refers to the perceived difficulty level that is required to perform a certain task. Individuals who expect a low-magnitude task often feel more confident than people expecting a high-magnitude task. *Strength* relates to an individuals’ judgment of their ability to perform a specific task. This dimension is pertinent to the individual’s strength of confidence and persistence in the face of frustrations and barriers. *Generality* is about the extent to which efficacy expectations can be generalized across situations. The three dimensions of self-efficacy are of great importance in developing the self-efficacy scale because they provide suggestions and implications for what elements should be evaluated.

### Translation Psychology

The scope of psychology ranges from cognition to emotion or affect and personality (Jääskeläinen, 2012). Translation psychology is a multifaceted construct, defined as “the subdiscipline of translatology that addresses the study of translators as complex individuals functioning as a whole” (Bolaños-Medina, 2016). In a narrow sense, it is about the translator’s psychological states in the process of translating, skill acquisition, and professional development (Zhu, 2020). The cognitive psychological approach focuses on probing the black-box of the human mind, involving thinking, perception, attention, emotion, and cognitive-related behaviors (Solso et al., 2005). Thinking is the most complex component of cognitive psychology. In translation, the translator’s mind cannot be directly observed. Translation thinking is related to the cognitive process of bilingual and bicultural understanding as well as transfer in the translation process.

Data-collection methodologies in translation can be process-, product-, participant‐ and context-oriented (Zhu, 2020). Different research tools are often adopted in terms of different oriented approaches. For instance, the process-oriented approach focuses on behavior observations by keystroke logging and eye-tracking, while the participant-oriented approach often employs questionnaires and interviews. A Likert-type of questionnaire can be used to explore the relationships among cognitive constructs and traits. A recent study by Schaeffer et al. (2020) developed a questionnaire to examine translation competence based on self-reported data. The reliability, validity, and quantitative analysis of the data obtained from a questionnaire can serve as a foundation for more rigorous cognitive translation research (Mellinger and Hanson, 2020).

## Prior Research on Translating Self-Efficacy

### Translating Self-Efficacy

Translation is closely related to language learning activities. A body of studies has revealed that self-efficacy can be a facilitator in the language learning process. For instance, Bai et al. (2019) conducted a study on 1,092 Chinese EFL learners and found that self-efficacy is positively correlated with English learning proficiency. Narrowing reading and writing research, belief in self-efficacy is found to be predictive of performance in reading (Shang, 2010) and writing (Sun and Wang, 2020). Since translation activities often involve reading and writing behaviors, we can postulate that self-efficacy might play a critical role in translating activities.

To discuss the role of self-efficacy in translation, it is necessary to mention “self-concept,” another belief close to self-efficacy but comparatively addressed more in translation studies (Kiraly, 1995; Heeb, 2016). According to Kiraly (1995), a translator’s self-concept includes “a sense of the purpose of the translation, an awareness of the information requirements of the translation task, a self-evaluation of the capability to fulfill the task, and a related capacity to monitor and evaluate translation products for adequacy and appropriateness.” Self-concept takes a central position in Kiraly’s psycholinguistic translation process model. This construct is also a part of Göpferich’s (2009) translation competence model, which influences how translators perform when translating. To determine participants’ focal points in translating, Ehrensberger-Dow and Massey (2013) conducted a study on the self-concept of translation students and translation professionals. They found that translation competence level may be associated with a translators’ self-concept development level.

Self-efficacy is considered to be one sub-dimension of self-concept (Kiraly, 1995; Muñoz Martín, 2014; Haro-Soler, 2019b). The limited literature indicates that self-efficacy has played a critical role in translation performance. Earlier research finds that self-confidence, a synonym often used for self-efficacy, can be considered as one of the prerequisites for creative translation (Kussmaul, 1995) and a facilitator for translation quality (Tirkkonen-Condit and Laukkanen, 1996). Subsequent research on self-efficacy suggests that self-efficacy can contribute to translators’ motivation and job ability (Atkinson, 2014; Bolaños-Medina, 2014; Haro-Soler, 2017). For example, Bolaños-Medina (2014) suggest that there are positive correlations among students’ self-efficacy, source language reading comprehension, and their ability to find background documentary information. A high level of translating self-efficacy can entail the management and computer-aided translating skills, while low translating self-efficacy may lead translators to spend much time in the translating process (Araghian et al., 2018). The growing recognition of self-efficacy in translation highlights the need of measuring translating self-efficacy.

### Measurement of Translating Self-Efficacy

To examine translating self-efficacy, translation competence should be first taken into consideration. From the product-perspective, translation competence is necessary to produce a good-quality target text in compliance with all the relevant norms followed by professional translators (Quinci, 2015). From the linguistic-perspective, translation competence is viewed as the summation competence working on reading and writing activities as well as transferring competence between two different languages (Gutiérrez, 2018). In any case, it should be noted that translation competence is a multicomponent construct.

Self-efficacy has been invariably mentioned in some dominant translation competence models. For example, Process of Acquisition of Translation Competence and Evaluation (PACTE, 2005) proposes a model comprising five sub-competencies (bilingual, extralinguistic, knowledge about translation, instrumental, and strategic sub-competence), as well as psycho-physiological components. Self-efficacy is one of the psycho-physiological components that can connect to the other five sub-competences.

Göpferich (2009) later developed a model of translation competence in a longitudinal study. This model consists of communicative competence in source and target language, domain competence, tools and research competence, translation routine activation competence, psycho-motor competence, and strategic competence. In this model, self-confidence and perseverance are considered elements of psycho-motor competence. Another influential competence model is proposed by European Masters in Translation (EMT, 2009) expert group. EMT competence framework is market-driven and practical, consisting of translation service provision, language, intercultural, information mining, technological and thematic competence. These models postulate dimensions of translation competence from different perspectives and with different highlights. Among these models, overlapping components can be identified, for example, bilingual communicative competence, instrumental competence, and a translator’s psycho-physical disposition. The commonalities have implications for the development of the translating self-efficacy scale in the present study.

A review of the literature indicates that there are many tools to assess belief in self-efficacy. For example, the General Self-efficacy (GSE) scale by Baessler and Schwarzer (1996) is a 10-item single-factor instrument. This scale has been translated into many languages and used in prior research (Bolaños-Medina, 2014). Despite the wide use of GSE, some researchers claim that in contrast to single-factor scales, multidimensional instruments can do better in assessing self-efficacy (Wang et al., 2013). Responding to this consideration, the three-factor Academic Self-efficacy (ASE) scale by Kim and Park (2001) is also frequently used in assessing college students’ academic self-efficacy. By referring to ASE, Lee (2014) develops an Interpreting Self-efficacy (ISE) scale, comprising dimensions of self-confidence, self-regulatory efficacy, and preference for task difficulty.

Although the above-mentioned scales have been proved to have solid psychometric properties, they are not domain-specific to translating self-efficacy. Bandura (2006) argues that self-efficacy measurement should be domain specific, indicating that domain-specific self-efficacy scales are more likely to have a stronger predicting power for specific behaviors than the ones for general use. The domain-specific guideline has called for efforts to incorporate translation elements into translating self-efficacy scale.

Two typical self-efficacy scales specific to translation were developed by Bolaños-Medina and Núñez (2018) and Haro-Soler (2018). The scale by Bolaños-Medina and Núñez (2018) is a 20-item five-factor scale, developed in a sample of 74 undergraduate translation students with English as the first language. The five factors are communicative/pragmatic competence, self-evaluation and learning, problem-solving, client-related issues, and strategic competence. This scale is tested by exploratory and confirmatory factor analysis but has overlooked the investigation of instrumental competence. The scale, designed by Haro-Soler (2018), is targeted for the language pair of English and Spanish. It consists of 25 items, covering beliefs regarding communicative and textual competence, cultural and intercultural competence, instrumental competence, interpersonal competence, strategic competence, and belief in working in the labor market. This scale was piloted on 176 students and has undergone rigorous content validity assessment by a panel of experts. The scale was used in translation teaching practices to monitor 39 students’ self-efficacy changes. However, there was no description of the scale factor analysis in the study.

The creation of the two scales has greatly narrowed the gap in translating assessment and contributed to understanding translators’ perception of the translation process. However, neither of them is targeted at student translators in a broad sense and the translation direction of English and Chinese. The sample size is also an essential factor in scale development. Small sample sizes can provide an inadequate representation of the intended population (Worthington and Whittaker, 2006). In addition, the perception of translating self-efficacy may vary across cultures. For example, it has been found that Asian students report lower self-efficacy beliefs than non-Asian peers (Scholz et al., 2002). The core translation competence of Chinese student translators is their language proficiency, specifically the English proficiency relevant to appropriate expressiveness (Yang, 2002). This statement is confirmed by Professor Li (2002) who conducted an empirical study on the learning needs of translation students. This study found that students are aware that they have much to improve in language proficiency. Furthermore, another Chinese translation scholar Professor Ma (2013) argues that the biggest problem facing Chinese student translators is their command of English expressions.

Taking the above into consideration, the purpose of the present study is to develop a reliable and valid translating self-efficacy scale (TSE-C) for non-English native speakers including translation students, language students, and non-language students who want to learn translation knowledge and could be potential translators in future. Exploratory factor analysis (EFA) and confirmatory factor analysis (CFA) were conducted in two independent samples (Sample I = 193 and Sample II = 247) to examine the factor structure and validity of the scale.

## Materials and Methods

### Participants

To minimize selection bias, participants were randomly invited from different universities in China and voluntarily participated in this study. They were native speakers of Chinese and took English as a foreign language. All of them were instructed with basic translation knowledge, theories, and skills. They were translation majors, English majors, and non-language majors. English majors and non-language majors received translation training for 6 months, and translation majors received 12 months of training. Since the purpose of the present study was to measure the psychometric properties of TSE-C, a self-efficacy level comparison among students with different translation experiences was outside the scope of the present study and was not considered.

The sample choice was made, based on the two main considerations. First, non-translation majors with excellent foreign language skills are often expected to undertake translation activities, especially specialized ones (Pan, 2018). For example, Namdari and Shahrokhi (2015) found that Iranian chemistry students who know English well outperform translation students in a chemistry text translation. Second, there was evidence that individuals without translation degrees are active in the translation service industry (Pérez-González and Susam-Saraeva, 2012). In China, in addition to graduates of translation majors, graduates of language majors and even non-language majors have also embarked on a career in translation career (Qu, 2002). According to Patton’s “maximum variation sampling” (Lincoln and Guba, 1986; Li, 2002), sample selection can allow for maximum variation in participants’ translation experience and educational background.

The overall sample size of the study was 440 based on accessibility. It was split into two groups under the guidelines of EFA followed by CFA using a different sample to evaluate the scale factor structure and psychometric properties (Henson and Roberts, 2006). Prior research suggested that a sample size between 100 and 200 was the minimum base for initial EFA and CFA (Worthington and Whittaker, 2006). Other guidelines indicated at least a 5:1 ratio of participants to the number of parameters (Bentler and Chou, 1987). Since the scale of TSE-C was targeted for translation learners in a broad sense, a heterogeneous sample of students was recruited in the present study. A large heterogeneous sample provides an item pool for consideration of structural validity (Clark and Watson, 1995). This sort of sample choice is often used in scale development research, for instance, the scale development of academic writing self-efficacy (Mitchell et al., 2021), computer self-efficacy (Harrison and Rainer, 1992), and anxiety disorder (Johnson et al., 2019). In this study, Sample I for EFA consisted of 193 second-year undergraduate students with 108 English majors and 85 non-English majors. The average age was 19.4 years old. The gender composition was 16% male and 84% female. Sample II for CFA consisted of 247 students. Of these, 89 were second-year non-English majors, 121 were second-year English majors, and 37 were translation students. The average age was 21.2 years old, and the gender distribution was 19% for male and 81% for female participants.

### Instrument Development

Scale development can proceed with item writing, pilot testing, and factor analysis to provide a sound basis for applied research (Mellinger and Hanson, 2020). To guarantee the validity of the proposed instrument and ensure that the items can exactly reflect the translating self-efficacy of Chinese student translators, we adopted the *Discriminant Content Validity* approach (Johnston et al., 2014), which has been successfully used in scale content analysis. The analysis consisted of the following four steps.

#### Defining the Construct

To clarify the attribution of self-efficacy and produce a working definition of translating self-efficacy, we first combed through literature on self-efficacy belief. The dimensions and sources of self-efficacy have been discussed in detail in past studies. Many studies framed self-efficacy as connected to a students’ self-judgment of their capability to complete a specific task. From this perspective, students’ self-judgments of their translation competence should be considered to assess their self-efficacy (Araghian et al., 2018). We therefore loosely interpreted translating self-efficacy as student translators’ self-perception of their competence when performing translation tasks.

#### Generating Items

After clarifying the definition of translating self-efficacy, we then tried to generate items by reviewing the literature about self-efficacy scale development and translation competence. To test belief in self-efficacy in translating, we referred to some self-efficacy scales for their pertinence to translation. These were the *Language Learning Self-efficacy Scale* for reading, listening, speaking, and writing (Wang et al., 2014), *Interpreting Self-efficacy Scale* (Lee, 2014), and the *Translating Self-efficacy Scale* by Bolaños-Medina and Núñez (2018) and Haro-Soler (2018). The translation competence model by PACTE (2005) was another reference for competence self-assessment consideration. PACTE group postulates that internal and external support are important in translators’ decision-making process. Internal support is based on the knowledge retrieved from a translator’s long-term memory, covering linguistic, extra-linguistic knowledge, and strategies. External support is about instrumental capability, including relevant documentation sources and the use of technological tools (Albir and Alves, 2009). The skills and knowledge associated with external support are referred to as the instrumental sub-competence by PACTE. The two supports to an extent, encompass the proposed sub-competencies in the PACTE’s model. Through carefully reviewing the literature on self-efficacy and analyzing the components of translation competence, we initially obtained a pool of 45 items for TSE-C.

#### Establishing the Scale

At this step, items were categorized based on their semantic content. For instance, items on the ability to identify problems and make inferences were merged into the internal support group. To assess participants’ perceived ability to carry out a behavior, items were phrased based on statement such as “I’m confident that” and “I can,” as suggested by Bandura (2006). These items were rated on a five-point Likert scale, ranging from strongly disagree (coded as one point) to strongly agree (coded as five points).

#### Testing the Content Validity

Content validity refers to the degree to which a test measures the content domain it purports to measure (Sireci, 1998). Content validity studies typically involve a relatively small number of participants who are required to make a variety of important judgments. It is suggested that scale items can be checked by professionals for content analysis and piloted by students for comprehensibility consideration (Xiong et al., 2014).

Before the TSE-C scale was formally presented to the participants in the present study, the content validity index (CVI) was measured to ensure the relevance and clarity of the items. Referring to a study by Polit et al. (2007), the items were assessed based on a four-point scale labeling from 1 (not relevant) to 4 (highly relevant). Three experts were invited to examine if the scale items were appropriate, relevant, and fit the theoretical model of translation competence. All are professionals in translation studies, with years of experience in translation teaching and practice. One of them is also an expert in educational psychology and data analysis. The experts were instructed individually with the scale copy. They were required to examine each item, match the item with its respective translating self-efficacy index, and assess the relevance between the item and the index. Furthermore, to check the item readability and comprehensibility for students, the scale items were also piloted by 10 Chinese students, including translation majors, English majors, and non-language majors in subjects like Computer science given the sample constitution. Experts’ judgments were then compared and calculated. It is suggested that items with CVI above. We considered there to be evidence of good content validity when 78 was awarded by three or more experts (Polit et al., 2007). Thus, the scale items with a CVI of 0.8 or above remained and the rest were discarded. Based on the suggestions and revisions from the experts and the piloted students, we finally refined and reduced the original 45 items into 21 items.

### Data Collection and Analysis

Online surveys have the allure of including potentially larger samples and can reach a diverse sample of respondents (Mellinger, 2015). The Chinese version of the present scale was presented to two different groups of participants through an online survey tool *Wen Juan Xing*^1^ in 2 weeks. Before the collected questionnaire data was uploaded to social statistical software SPSS for systematical analysis, each participant’s response was carefully screened. The questionnaire response time was estimated to be approximately 8–10 min. Those who took a too short or too long time to complete the questionnaire were excluded from the database. Therefore, 193 remained from 200 participants for the first-time questionnaire distribution, and 247 remained from 250 participants for the second-time distribution.

A *t-test* was used to evaluate the discrimination capacity of each item based on the levels of violence (Penagos-Corzo et al., 2019). EFA was conducted with SPSS 17.0 to discover the scale factor structure. For the factor extraction process, the main criteria were eigenvalues higher than 1 and factor loadings higher than 0.40 (Gratz and Roemer, 2004). CFA was carried out on AMOS 22.0 to confirm the factor structure. Convergent, concurrent, and discriminant validity were considered. Reliability was established by an internal consistency method using Cronbach’s Alpha.

## Results

### Exploratory Factor Analysis

Discriminant analysis of items was carried out in two groups of participants by comparing the 25% highest scores with the 25% lowest ones. A *t-test* achieved a level of significance (*p* < 0.05). All the items showed significant differences between groups and no item had to be removed. Bartlett’s test of sphericity and the Kaiser-Meyer-Olkin measure of sampling adequacy was first checked to assess the scale factorability. The KMO value was 0.89 and Bartlett’s Test of Sphericity was significant (*p* < 0.001), which indicated that items in TSE-C were appropriate for factor analysis.

**Figure 1:**
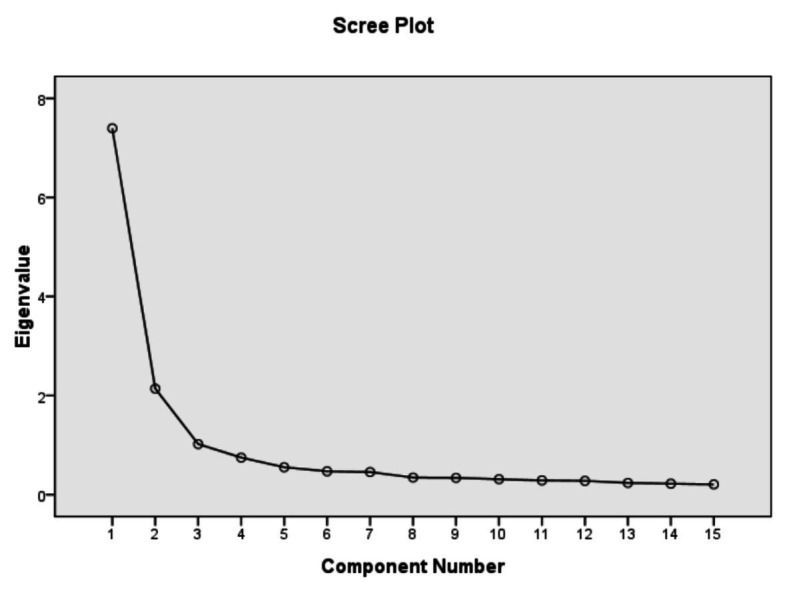
Scree plot of TSE-C.

To identify the factor structure of TSE-C, EFA was performed by using the principal axis factoring method with promax rotation so that the underlying factors could be correlated. Kaiser’s criterion (retaining factors with eigenvalues greater than 1) and parallel analysis were both considered in determining the number of emergent factors to extract. In the first round of EFA, six items were deleted as they had a cross-loading problem with a loading <0.4 on any factors. The other 15 items were retained and the EFA yielded a three-factor structure. The scree plot of the scale is presented in Figure 1.

To cross-validate the three-factor structure obtained in the first round EFA, a second round EFA, with a refined 15-item scale, was then performed using parallel analysis. In a parallel analysis, raw data with eigenvalues greater than those in the random data were retained. The results indicated that three eigenvalues from the raw data were greater than those from random data created by the Monte Carlo Simulation, also suggesting a three-factor structure of the scale. The factor loading of each item is shown in Table 1.

**Table 1:** Factor loadings and values for Cronbach’s alpha.

Constructs/items	Factor loading	α
Factor 1: Efficacy in Internal Competence (EIC)		0.91
BC1: I can identify subtle word meaning differences.	0.65	
BC2: I can gain a better understanding of the text than my peers.	0.74	
BC3: I can translate the source text correctly and fluently.	0.81	
EC1: I can understand the social and cultural factors in the source text.	0.71	
EC2: I can discern ideological differences in two cultures.	0.73	
EC3: I can use appropriate words in translation.	0.77	
SC1: I can quickly identify translation problems and make decisions.	0.77	
SC2: I can make inferences and draw conclusions in translation.	0.82	
SC3: I can choose appropriate translation strategies in translation.	0.65	
Factor 2: Psycho-physiological Components (PPC)		0.81
PS1: I can improve my translation ability through continuous efforts.	0.89	
PS2: I can work hard to become a competent translator.	0.59	
PS3: I can self-evaluate the translation integrity and appropriateness.	0.89	
Factor 3:Efficacy in External Competence (EEC)		0.79
IC1: I can overcome difficulties in translation technology use.	0.62	
IC2: I can make full use of the digital resources to search information.	0.60	
IC3: I can take readers’ needs into consideration in translation.	0.67	

The resulting factor structure analysis revealed the presence of three distinct factors. Nine items loaded on Factor 1 and each had high loadings ranging from 0.65 to 0.82 without any cross-loading items. Further observations found that items on this factor were associated with internal knowledge about translation competence, including linguistic competence (BC1, BC2, and BC3), extralinguistic competence (EC1, EC2, and EC3), and translation strategies (SC1, SC2, and SC3). Therefore, Factor 1 was named as *Efficacy in internal competence* (EIC). Factor 2 consisted of three items with loadings from 0.59 to 0.89. The three items were regarding translators’ *Psycho-physiological components* (PPC), including effort (PS1), perseverance (PS2), and self-evaluation (PS3). The remaining three items loaded on Factor 3 with loadings of 0.62, 0.60, and 0.67, respectively. The three items generally addressed the external competence of translators, including coping with difficulties (IC1), manipulating digital resources (IC2), and translation service (IC3). Factor 3 was thus named as *Efficacy in external competence* (EEC). The factor loadings of all items were higher than the acceptable value of 0.40 (Field, 2005) and the extracted three factors together accounted for 56.46% of the common variance, with Factor 1 for 40.67%, Factor 2 for 10.30%, and Factor 3 for 5.49%. Reliability analysis indicated that the instrument had a strong internal consistency with Cronbach alpha value of 0.90 for TSE-C, 0.91 for EIC, 0.81 for PPC, and 0.79 for EEC, indicating good internal consistency.

### Confirmatory Factor Analysis

#### Factor Analysis

To replicate the factor structure identified through EFA, CFA was conducted with Sample II (*N* = 247), a different sample of participants. The Cronbach’s alpha value of TSE-C in Sample II was 0.93 and the values for subscale EIC, EEC, and PPC were 0.93, 0.70, and 0.81 respectively, which suggested good internal consistency. The descriptive statistics of Sample II indicated that the skewness and kurtosis value of each item was from 0.02 to −0.81 (<2) and from 0.45 to 2.36 (<7) respectively, suggesting maximum likelihood (ML) was suitable to estimate the parameters and verify the factors (Xiong et al., 2014). According to Kline (2011), the model fit was assessed using several indices: chi-square statistics (*χ*^2^/df < 3), comparative fit index (CFI ≥ 0.90), the Tucker-Lewis index (TLI ≥ 0.90), the Root Mean Square Error of Approximation (RMSEA < 0.08), and Standardized Root Mean Square Residual (SRMR ≤ 0.10). Model modification decisions were based on item psychometric considerations and scale content. Standardized factor loadings lower than 0.40 were removed.

The CFA results showed all standardized factor loadings were statistically significant (*p* < 0.001) with values from 0.59 to 0.85. However, the initial model fit indices were not satisfactory (*χ*^2^/df = 3.27; CFI = 0.92; TLI = 0.90; RMSEA = 0.09; SRMR = 0.07). Inspection of the model modification indices demonstrated that residual covariance involving EC1 and EC2, BC1, and BC2 could contribute to improving the model fit. Item EC1 and EC2 were relatively similar in wording and specified to measure the same construct. The suggested correlation between item EC1 and EC2 was theoretically justifiable because both items involved the measurement of cross-cultural competence. Likewise, BC1 and BC2 were similar-worded items and purposely developed to measure the same construct. It was recommended that similar-worded items could induce error correlations between indicators (Brown, 2006) and correlations to account for similar-worded items were not problematic (Schwartz et al., 2012). After the step-by-step modifications, the final Model fit indices indicated the model had acceptable fit indices (*χ*^2^/df = 2.30, CFI = 0.95; TLI = 0.94; RMSEA = 0.07; SRMR = 0.07). See Table 2.

**Table 2:** Model fit indices for the TSE-C.

	Model	Acceptable values
*χ*^2^/df	2.3	<3
CFI	0.95	≥0.90
TL1	0.94	≥0.90
RMSEA	0.07	<0.08
SRMR	0.07	≤0.10

#### Convergent Validity

Convergent validity refers to whether indicators from a latent variable do belong to that latent variable (Wang et al., 2015). To evaluate the convergent validity, several indices are suggested: factor loading (≥0.5), composite reliability (≥0.7), and average variance extracted (≥0.5) (Fornell and Larcker, 1981; Hair et al., 2010). From Table 3, it can be concluded that values of CR and AVE were both acceptable, which indicates that the convergent validity of the scale is good.

**Table 3:** Convergent validity for the model.

Construct/item	FL(λ)	RC (λ^2^)	EV(1-λ^2^)	CR	AVE
EIC				0.93	0.60
BC1	0.63	0.40	0.60		
BC2	0.71	0.50	0.50		
BC3	0.85	0.72	0.28		
EC1	0.81	0.66	0.34		
EC2	0.75	0.56	0.44		
EC3	0.78	0.61	0.39		
SC1	0.81	0.66	0.34		
SC2	0.82	0.67	0.33		
SC3	0.79	0.62	0.38		
PPC				0.81	0.58
PS1	0.75	0.56	0.44		
PS2	0.76	0.58	0.42		
PS3	0.78	0.61	0.39		
EEC				0.78	0.54
IC1	0.81	0.66	0.34		
IC2	0.74	0.55	0.45		
IC3	0.64	0.41	0.59		

FL, factor loading; RC, reliability coefficient; EV, error variance; CR, composite reliability; AVE, average variance extracted.

#### Concurrent Validity

Concurrent validity is about the degree to which a measure is associated with measures of similar content (Hewitt et al., 1991). In this paper, the concurrent validity of the scale was assessed against scores on the scale of GSE (Schwarzer and Jerusalem, 1995), an external measure of constructs relevant to TSE-C. In Table 4, TSE-C was positively and significantly correlated with GSE (*r* = 0.40). Its sub-scales were also positively and moderately linked to GSE. The correlation results suggest an acceptable concurrent validity of the scale.

**Table 4:** Concurrent validity of the scale.

Scale	GSE
TSE-C	0.40^∗∗^
EIC	0.34^∗∗^
EEC	0.25^∗∗^
PPC	0.33^∗∗^

^∗∗^Significant at 0.01 probability level (*p* < 0.01).

#### Discriminant Validity

Discriminant validity differentiates one construct from another in the same model (Wang et al., 2015). According to Hair et al. (2010), the correlation of two latent constructs is over 0.9, suggesting significant overlappings of the constructs. In the present study, the correlation values among the latent constructs are all below 0.9, to be specific, 0.80 for (PPC-EEC), 0.63 for (EEC-EIC), and 0.52 for (PPC-EIC). The findings also suggest that efficacy in internal competence and external competence are mutually affected. Compared with internal competence, external competence demonstrates a stronger relationship with psycho-physical components. By and large, supporting evidence from research findings demonstrates that the proposed TSE-C tool has good reliability and validity properties.

## Discussion

This article falls within the framework of translation psychology which covers the skill acquisition and psychological analysis of the translator’s mental operations. Self-efficacy, being a psychological factor, is believed to have a close relationship with translation performance. The crucial role of self-efficacy in translator education has gathered effort to establish a translating self-efficacy tool. This study developed a translating self-efficacy scale from a sample size of 440 students, consisting of translation students, English majors, and non-English majors. EFA (Sample I = 193) and CFA (Sample II = 247) were conducted to cross-validate the properties of scale items. The results of EFA suggested a three-factor structure, including efficacy in internal competence, efficacy in psycho-physical competence, and efficacy in external competence. CFA was conducted to verify the identified model. Convergent, concurrent, discriminant validity, and reliability analysis provide supporting evidence for instrument use.

Compared with the scales by Bolaños-Medina and Núñez (2018) and Haro-Soler (2018), the developed TSE-C scale is special in a number of ways, outlined below.

### Sample Constitution

TSE-C scale is targeting student translators in a broad sense, particularly those in English as a foreign language environment, including Chinese translation majors, English majors, and non-language majors. However, the scales by Bolaños-Medina and Núñez and Haro-Soler are targeting student translators in a narrow sense, that is, the translation students. The participants in the study by Bolaños-Medina and Núñez have English as the first language, and the participants in the study by Haro-Soler take Spanish and English as translation language pairs. Since the nature of respondents can influence the structure and properties of a scale, the measurement provided by the scale can be presumed to be valid and reliable only for respondents similar to the original ones used in the scale development (Mellinger and Hanson, 2020). Nevertheless, translation is a highly attractive career for young people with a love for languages and for engaging with other cultures (Baker, 2018). The translator community is not only made up of translation graduates but language or even non-language graduates in the translation industry (Rennie, 1979; Qu, 2002). Therefore, the mixing constitution requires notice of the invisibility of non-translation students in translator education.

### Scale Development Approaches

Factor analysis is an essential step for initial scale development to complete full psychometric testing (Mellinger and Hanson, 2020). It consists of EFA and CFA to ensure the scale reliability and validity using a sample size usually over 100 (Worthington and Whittaker, 2006). EFA is essential in statistically identifying the factor structures at the early stage of scale development and CFA is necessary to support the final validity (Beaujean, 2014). In terms of the pioneering two scales, one is generated based on EFA and CFA in a sample of 76 students and the other does not add much description to the factor analysis. Because of this, the TSE-C scale was tested and validated through two independent samples in EFA and CFA to make up for the inadequacy.

### Factor Structures

The scale by Bolaños-Medina and Núñez (2018) is a five-factor structure of translating self-efficacy, including communicative/pragmatic competence, self-evaluation/learning, problem-solving, client-related issues, and strategic competence in self-efficacy analysis. Haro-Soler (2018) classifies translating self-efficacy belief into six dimensions, regarding communicative and textual competence, cultural and intercultural competence, instrumental competence, interpersonal competence, strategic competence, and belief in the ability to translate specialized texts in the translation market.

Based on the scales by Bolaños-Medina and Núñez (2018) and Haro-Soler (2018), TSE-C is a concise measure of three-factor structure, including efficacy in internal competence, efficacy in psycho-physical competence, and efficacy in external competence, which puts more emphasis on bilingual and strategic competence. Students believe that when they have acquired much better bilingual competence they start translation (Li, 2002). The bilingual and translation knowledge competence are also the focus in translator trainers’ training practice (Wu et al., 2019). Altogether, it can not be denied that the fruitful work by Bolaños-Medina and Núñez (2018) and Haro-Soler (2018) has contributed significantly to the translating self-efficacy instrument development. Same as the two other pioneering scales, it should be acknowledged that the TSE-C scale is not universally valid but rather a preliminary one.

Responding to the critical role of self-efficacy in translation and the mixing constitution of the translator community in the language service industry, this study is of significance in translator education. It is believed that investigations on students’ translating self-confidence are useful for understanding the teaching approach values (Kiraly, 1995). TSE-C can be applied as a measure to inspect students’ perception and self-judgment of their translating competence. For example, TSE-C could help identify low-efficacious students. Through reflecting on the translation learning process, the low-efficacious students can address unproductive strategies under the guidance of teachers. High-efficacious students may be more self-confident and more likely to be responsible during the translation process. The self-efficacy level can serve as a clue for students in identifying specific areas that require improvement. Furthermore, understanding students’ translating self-efficacy is crucial for the design of translation courses. For instance, students’ self-efficacy information in processing translation tasks may provide suggestions and implications for teachers to modify their teaching strategies.

## Conclusion

This study developed a reliable and valid translating self-efficacy scale TSE-C to assess the translating self-efficacy of student translators. TSE-C was embedded for translation learners in an English as a foreign language environment. The scale was of adequate quality to be recommended for translation teaching and research purposes. Although promising results were obtained, it is important to bear in mind that the application of TSE-C should be approached with caution and some limitations need to be addressed. First, this study was cross-sectional and the findings were solely drawn from self-reported data. Interview data may be helpful to triangulate the scale validity. Second, the study did not test the predictive power of TSE-C, and the stimulating nature of translating self-efficacy was not confirmed in translation teaching and learning activities. Third, the interpersonal skill of student translators has been overlooked, which is necessary for becoming a successful translator in the language service industry. In the future, we will collect interview data after the application of TSE-C in translation activities and investigate the relationship between students’ translating self-efficacy and their performance. The role of interpersonal skills will be considered in interpreting students’ translating self-efficacy.

## Data Availability Statement

The raw data supporting the conclusions of this article will be made available by the authors, without undue reservation.

## Ethics Statement

The studies involving human participants were reviewed and approved by the Ethics Committee of Nanjing Agricultural University. The patients/participants provided their written informed consent to participate in this study.

## Author Contributions

YY generated the initial idea, analyzed/interpreted the data, and wrote this manuscript. XC revised this manuscript substantially. XH provided suggestions for the improvement of the manuscript. All authors contributed to the article and approved the submitted version.

### Conflict of Interest

The authors declare that the research was conducted in the absence of any commercial or financial relationships that could be construed as a potential conflict of interest.

## Footnotes

^1^https://www.wjx.cn
